# Training Healthcare Professionals to Deliver a Group‐Based Intervention for People Living With Severe Obesity: Lessons From the PROGROUP Feasibility Trial

**DOI:** 10.1111/jhn.70204

**Published:** 2026-01-28

**Authors:** Shokraneh Moghadam, Mark Tarrant, Lily Hawkins, Dawn Swancutt, Rod Sheaff, Laura Hollands, Raff Calitri, Jonathan Pinkney, Jenny Lloyd

**Affiliations:** ^1^ University of Exeter Exeter UK; ^2^ University of Plymouth Plymouth UK

**Keywords:** group‐based interventions, healthcare professionals, obesity, training, weight management

## Abstract

**Introduction:**

Group‐based programmes are increasingly adopted to support people living with obesity, as they have the potential to reduce staff time and costs and enhance motivation and capability for behaviour change. Group‐based programmes could also provide members with opportunities to form meaningful social connections. As such, training should equip healthcare professionals to deliver group interventions successfully and be accessible and feasible for them to complete. This study presents how mixed‐methods process evaluation data from the feasibility randomised controlled trial of PROGROUP (a group‐based weight management intervention for people living with severe obesity) informed optimisation of the PROGROUP training programme.

**Methods:**

Five healthcare professionals (facilitators) from three specialist weight management services across the United Kingdom participated in a 4‐day remote training programme in preparation for facilitating PROGROUP. Four patient cohorts were subsequently delivered across these services. Training content was informed by psychological theory and included communication and group facilitation skills, as well as physical activity and dietary education, delivered by an expert research and practitioner team. Following intervention delivery, facilitators were interviewed about their experiences of training and delivery. Additional data collection sources included fidelity (to form and content) checklists, audio and video recordings of intervention delivery, and field note summaries from in‐person observations and debrief calls. Interview data was analysed thematically.

**Results:**

Fidelity findings showed that intervention content was largely delivered as intended across all cohorts (average across cohorts = 68%), with facilitators showing confidence in delivering the educational components of the intervention. However, variability was observed across cohorts, indicating differences in facilitators' ability to deliver flexibly and in line with intervention delivery principles. Facilitators also reported challenges in attending all training days in full and expressed a preference for more self‐directed learning. In response, to improve accessibility and delivery fidelity, training was adapted to include self‐directed learning and a greater focus on developing the necessary skills and knowledge to facilitate according to intervention delivery principles.

**Conclusion:**

This study outlines a process for optimising training for healthcare professionals, with learnings applicable to other group‐based healthcare settings. Findings highlight the need for healthcare professionals to develop skills aligned with delivering such interventions with fidelity, to maximise potential effectiveness. A flexible, self‐directed training format can enhance feasibility and acceptability, balancing healthcare professionals' needs with intervention requirements, and offers a valuable and novel training resource for those delivering group‐based care.

## Summary

- Group‐based interventions could enhance behaviour change in people living with obesity, particularly when patients develop meaningful social connections. Fostering these connections could help make behavioural changes more sustainable.
- Training should support healthcare professionals to confidently facilitate group sessions in ways that promote shared social identity amongst members, while maintaining fidelity to the intervention structure. Flexibility in training delivery is also essential to accommodate practitioners' work schedules.
- Group facilitation skills are widely transferable, enabling practitioners to effectively support patients in weight management and other behaviour change programmes.

## 1 Introduction

In the United Kingdom, an increasing number of specialist weight management services are adopting a group‐based model of care to support behaviour change in people living with severe obesity (PLWSO) [1]. Group‐based behaviour change programmes may address pressures caused by a combination of growing waiting times, potentially exacerbated by the Covid‐19 pandemic [2], and individual consultations, but they may also provide wider therapeutic benefits for patients that arise from developing a sense of social identity that is shared with other members of the group [3]. Shared social identity may also empower patients to use the group as a resource that motivates and sustains their engagement with the programme's behaviour change content [3]. For example, interview data from PLWSO, attending a group‐based weight management programme, suggested that shared social identity underpinned engagement with the intervention [4]. Participants in that study also described how identifying with others in the group underpinned their motivation to engage with behaviour change content and supported them in making desired behavioural changes. Such shared identity could help people living with obesity to feel less alone and potentially enhance their self‐efficacy to make change, for example, through mutual support and learning from others' experiences. However, such outcomes may only be achieved if healthcare professionals (HCPs) have the necessary knowledge and skills to create the conditions for members to experience the group in this way. Therefore, training in the skills and knowledge to foster shared social identity within groups, while simultaneously delivering the intervention's behaviour change content, is essential to maximise the potential of group‐based care.

All interventions that aim to change behaviour are, by nature, complex, as they consist of a combination of components and delivery approaches (forms) [5] to achieve their function. Arguably, group‐based interventions involve an added layer of complexity, which arises from the interactions amongst group members, and between group members and group facilitators [6, 7]. This complexity presents challenges when developing feasible and acceptable training for HCPs, as such training is rare and must prepare them to deliver the intervention effectively while managing the dynamics of group delivery. It requires that researchers assess and synthesise the experiences of training and intervention delivery, as well as fidelity to the form of the intervention, to understand whether training is fit for purpose. If training is not fit for purpose, it should be modified to better equip HCPs with the skills and knowledge to deliver interventions more effectively (i.e., to achieve the intervention's function).

The current study reports on a feasibility trial of a group‐based behaviour change intervention called PROGROUP (outlined below). Mixed‐methods data were collected as part of the process evaluation with the aim of assessing the acceptability and feasibility of the facilitator training programme and fidelity to delivery. How these data informed refinement of the training programme is presented in this paper. While the focus of this paper is on optimising training for HCPs delivering the PROGROUP intervention, the findings are intended to be generalisable to other healthcare contexts where group‐based care is planned.

### 1.1 The PROGROUP Intervention and fRCT

PROGROUP is a group‐based behaviour change intervention for PLWSO, designed for delivery by National Health Service (NHS) specialist weight management services in the United Kingdom [8]. The intervention consists of 12 group sessions and 3 one‐to‐one consultations and is delivered in‐person across a 5‐month period. The PROGROUP intervention comprises educational content on diet (e.g., the ‘eat well’ guide [9]), physical activity (e.g., becoming more active), and psychology (e.g., addressing negative self‐talk), along with behaviour change strategies, such as goal setting, for weight management. The intervention is informed by the Social Identity Model of Behaviour Change (SIM:BC) [3] and, in accordance with this, is designed to support patients to develop a sense of shared social identity, *as members* of the intervention group, in order that the group becomes a resource underpinning motivation and capability (including self‐efficacy and understanding) to pursue behaviour change. The core function of PROGROUP, therefore, is to create a sense of shared social identity amongst group members. The form of the intervention has been designed with this function in mind, providing the tools, including structured group activities, and a core set of delivery principles (the PROGROUP principles, see Appendix A) necessary to achieve a shared social identity amongst members (see [10] for a report of the intervention development process).

The feasibility randomised controlled trial (fRCT) of PROGROUP [8] was undertaken between June 2022 and August 2023 and involved 94 patients across four cohorts randomised to receive either PROGROUP (*n* = 47) or usual care (*n* = 47), in three UK specialist weight management services (Midlands = 2 cohorts, Wales = 1 cohort and Southwest = 1 cohort). Five HCPs (also referred to as facilitators) working as healthcare practitioners (dieticians and physical activity specialists) across the three specialist weight management services completed training to deliver the intervention.

### 1.2 The PROGROUP Training Programme

The PROGROUP training programme was initially planned for 5 days of face‐to‐face delivery; however, the Covid‐19 pandemic delayed the fRCT and necessitated a shorter training programme comprising 4 days of remote training via Zoom (see Supplementary Information S1). The training was delivered across a 5‐month period. An additional ‘catch‐up’ day was offered for HCPs who missed any of the training sessions. The training programme was delivered by members of the PROGROUP research team and an external practitioner team specialising in training weight management practitioners to support patient behaviour change. Training covered underpinning behaviour change theories and principles (e.g., SIM:BC), physical activity and dietary education, and activities (with individual feedback) to develop communication and group facilitation skills (e.g., role play) to build confidence and competence.

## 2 Methods

### 2.1 Data Collection

Mixed‐methods data were collected to assess delivery fidelity across cohorts (i.e., whether facilitators were able to deliver the intervention content flexibly, in order to meet the group's needs, and in accordance with the PROGROUP principles), understand any barriers to delivering the intervention as designed and assess the feasibility and acceptability of the training programme. Data sources included online semi‐structured interviews with facilitators post intervention delivery (*n* = 5) (Appendix B), conducted by a researcher on Microsoft Teams, and audio recorded using a dictaphone, a delivery checklist for manual content (Appendix C), completed after each session by the facilitator, and a delivery checklist for the PROGROUP principles (Appendix A), completed by the researchers using selected audio and video data (beginning, middle and end of the programme) (sessions 1, 2, 9 and 11) from two sites (Wales and Southwest), as well as weekly debrief calls with the facilitators to track and document any delivery issues (*n* = 20) (see Appendix D for how these outcome measures align with the study aim). An information sheet was shared with facilitators prior to interviews, and verbal consent was obtained on the day of the interview. Consent statements were signed and dated on behalf of facilitators by the interviewing researcher. Interviews lasted for approximately 1 h.

Reliability and accuracy of facilitator self‐assessed delivery of manual content was assessed using real‐time researcher observation of seven sessions across the three sites.

### 2.2 Data Analysis

An average percentage of activities fully delivered (i.e., all intended manual content was fully delivered) across sites and by individual sites was calculated to assess fidelity of delivery to manual content (Appendix C). Fidelity to the PROGROUP principles was discussed and agreed upon by researchers following individual assessments of the video and audio data using the checklist (Appendix A).

Facilitator interviews were transcribed by the researchers, using targeted transcription, and uploaded to the NVivo software (QSR International; Version 12 Pro). Qualitative data were analysed thematically [11], using the framework approach to data management [12, 13]. Members of the research team (*n* = 5) independently read and coded line‐by‐line a sample of the data and discussed and developed an agreed coding framework. New codes were reviewed as remaining transcripts were coded. Codes were then defined and organised into categories and themes, and these then informed modifications to the training programme. Field note summaries from debrief calls with facilitators were used to check whether they corroborated the interview and delivery fidelity findings.

### 2.3 Data Synthesis

Mixed‐methods data were integrated using a convergence coding matrix (Supplementary Information S2) to display key themes and findings associated with delivery fidelity, feasibility and acceptability of the training programme. Patterns or contradictory findings within and across sites were discussed with the core team (*n* = 9) over several meetings to understand factors affecting delivery fidelity and their implications for optimising the training programme (reported here: Table 1) and the PROGROUP intervention (Hawkins et al., in press).

## 3 Results

Findings showed that, overall, the majority of the intervention's manualised activities were delivered across all cohorts. However, there was variability in how flexibly facilitators adapted the intervention content to meet the needs of their group using the PROGROUP principles (Appendix A). Facilitators valued the peer learning approach to training but reported that certain barriers, such as working patterns, hindered full participation. Taken together, these findings indicated that the training programme required some *content* modifications in order to better support facilitators in delivering the intervention with fidelity. Changes to the *training format* were also needed to align with facilitators' other commitments, thereby enhancing training feasibility and acceptability. These findings, and how they informed the optimisation of the training programme, are presented below.

### 3.1 Training Format

Facilitators valued the online training for its ability to bring them together to share learnings and their experiences of delivering PROGROUP:

…I knew that there were [other] people who were going to be delivering the same and it's interesting to hear how other people delivered the stuff [intervention activities]….

F05

However, some facilitators expressed a preference for in‐person training, recognising that this would allow them the opportunity to meet face‐to‐face, but they acknowledged the downsides of this format:

…when you're training on one screen you lose an awful lot of interactions, compared to it being in the one room in the flesh, you know, trainers and the facilitators, especially the nature of this training … you're bound to pick up a lot of tips and ideas from your peers as well as trainers, but that's the pros and cons—there was no travel involved….

F03

In terms of attendance, attending the training days in full was challenging for most facilitators due to their limited availability, and for some, it clashed with their busy working patterns:

…training happened in the middle of my working week so…. I didn't have a lot of prep time in advance of training—I was doing a lot of it on the fly, you know….

F03

Instead, facilitators preferred a more self‐directed approach to training, one that allowed them the opportunity to complete parts of training in their own time:

[intervention training] needs to be a bit more achievable in terms of attendance and I think one of the things we discussed … was having some static content that people can go through in their own time and then use time together to do more of the practical stuff….

F04

These findings suggested that a more self‐directed and flexible approach to learning was preferred to enhance the acceptability and feasibility of training. Further details on the optimisation of the training format are presented in Section 3.3.

### 3.2 Training Content

In terms of fidelity to delivering the intervention content in line with the manual, findings from the self‐reported checklists showed that facilitators delivered the majority of the intervention's manualised activities (average across cohorts = 68%). However, the delivery of the manualised activities varied across cohorts, ranging from 59% to 80%. This variability was evidenced through fidelity to content checklists and showed that while some facilitators followed the manual rigidly, others demonstrated greater flexibility in content delivery in order to retain its alignment with the intervention's intended functions. Specifically, those who delivered the intervention flexibly tended to prioritise activities designed to promote a sense of shared social identity (e.g., a social network mapping activity) and the needs of the group, alongside or over content that was less relevant to the function of PROGROUP (e.g., a physical activity educational component). Accordingly, when facilitators stuck rigidly to the manual, intervention delivery tended to be more didactic:

…what happened was I just ended up going into like delivery, or teacher mode almost, which I know personally as a facilitator like it's not that helpful to have someone tell you stuff for an hour and a half….

F01

Such a didactic approach to intervention delivery, combined with rushed delivery due to time pressures, often led to missed or incomplete activities, particularly around group‐building tasks. This approach compromised the ability of facilitators to deliver in accordance with the PROGROUP principles, such as highlighting similarities between group members. These missed opportunities to connect and foster a shared social identity amongst group members often led to less engaged and well ‘gelled’ groups. For example, debrief call summaries showed that some facilitators struggled more than others at making the group ‘gel’, which consequently resulted in a group that was less well‐formed. One facilitator described their group as missing a ‘friendship element’ and feeling like a ‘mismatch’ of people:

…yeah I feel like there was a friendship element to cohort 1 and there wasn't a friendship element to cohort 2, it was just—there was a group of people that were put together that perhaps were a bit of a mismatch.

F05

In contrast, facilitators who were more confident in knowing how and when to deliver the intervention flexibly, and in line with the PROGROUP principles, encouraged group members to bond and connect with one another:

…you decide about well, what I can see happening here is people bonding, sharing or encouraging each other, anything based around getting the maximum value from the social side of things….

F04

For these facilitators, there was evidence to suggest that the patient group members were well connected and supportive of each other, especially during difficult conversations, such as those focused on life stressors. For example, one facilitator recalled group members ‘rallying’ around a participant who shared a distressing life experience, which in turn brought the group closer together:

…everybody in the group rallied around her and picked her back up … it was like a weight lifted from them so that—those two for me are the things that really brought those groups—that group—close together.

F02

This variability in the confidence of facilitators to deliver the intervention flexibly, and in line with PROGROUP principles, depended on their previous experience of delivering group‐based interventions, as well as their understanding of the needs of their group and its members at any given point in time. For example, one facilitator reported having the confidence to deliver group sessions according to a manualised plan, but soon realised the importance of a flexible and ‘empathetic’ delivery style in helping the group to connect:

I think definitely in terms of confidence of being in front of/at the front of a room leading a session working from a plan in the first session I did probably try to lean too heavily on those skills, whereas it was actually useful to be a bit more empathetic and let them [the group] talk as opposed to me talk….

F02

Despite variability in confidence, most facilitators were highly skilled in delivering the educational components of the intervention (i.e., physical activity and dietary educational components) and found little value in being trained on these topics:

…it was training around what you already do which was a bit hard to see the value…. I don't think we needed to practice facilitating the healthy eating stuff because that's like “I've heard all these before”….

F01

Instead, it was clear that more training on skills to deliver the intervention flexibly and in line with the PROGROUP principles should be prioritised in order to better equip facilitators to deliver the intervention as designed (i.e., with fidelity). For example, one facilitator reported having confidence in adapting the intervention content in line with the group's needs as being key to their delivery of the intervention, highlighting the importance of training that builds these skills:

…also having the confidence to look at the content and say, with this group and their particular challenges and goals and the journey we're at the moment, this bit of content isn't useful and actually we will spend the time doing something else. I don't know how you would build that up through a training programme.

F04

Similarly, other facilitators reported wanting more training on delivering behavioural skills (e.g., ‘action planning’) to groups of patients, in order to prepare them for delivering these techniques to PROGROUP members:

…we're talking about being more physically active, and action planning, and what not, that might be one of those techniques, to maybe teach the facilitators some more information around that and ways to implement in the group.

F02

Lastly, facilitators reported that more training in delivering the intervention's group‐building activities and managing group dynamics was needed, as these were skills in which they had little to no prior training. For example, more training in facilitating the ‘group pulse’, which provides facilitators the opportunity to check in with the group and encourage support amongst members:

…things like the group pulse, that's not something I've ever done before and it's always helpful to go over managing someone who's being challenging in a group. More of the group dynamics stuff, I feel there should've been more of a focus on that….

F01

### 3.3 Optimisation

**Figure 1:**
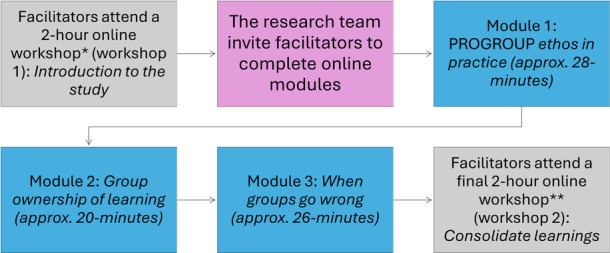
Optimised training pathway. Colour blue = self‐directed components; pink = administration; grey = online workshops. * Workshop to welcome facilitators, outline the ethos of the intervention and study, and focus on strategies to build shared social identity in weight management groups. ** Workshop to practice delivering a selection of PROGROUP sessions, in line with the intervention's delivery principles.

The above findings highlighted the need to adapt the training content to focus more on achieving the core function of PROGROUP, and the delivery format, to address facilitators' learning preferences (see Table 1). Specifically, changes were needed to the training content to provide more focused training on delivering the intervention flexibly and in line with the intervention principles. Additionally, the training format needed adaptation to allow facilitators to complete parts of the training in their own time through self‐directed modules. A workshop was convened with clinical members of the research team to discuss the implications of the findings for training and to agree on modifications (detailed in Supplementary Information S2). The optimised training pathway is shown in Figure 1.

**Table 1:** Summary of key limitations and modifications to the training format and content.

Training limitations	Modifications in response to findings	Purpose of modifications
Format		
Attending the training days in full was challenging for most facilitators due to their limited availability. Facilitators preferred a more self‐directed approach, allowing them the opportunity to complete parts of the training in their own time.	– The training format has been modified to include two (2‐h) online workshops and three web‐based self‐directed modules. – The 2‐h workshops are placed at the start of the training period and at the end (see Figure 1). Two hours for each workshop was deemed appropriate as it allows enough time to deliver necessary training content while remaining short enough to suit facilitators' availability. – The self‐directed modules are hosted on FutureNHS [14], and facilitators are invited to join this space once they have attended the first workshop. – The three online modules are delivered via short, interactive and informative videos, each followed by a brief quiz to assess understanding of the module content. – The modules are completed in facilitators' own time and take approximately 1–2 h to complete. Facilitators have approximately 1–2 weeks to complete the online modules before attending Workshop 2. – The online platform includes a forum to encourage facilitators to connect and share experiences of intervention delivery.	The training format was modified to a self‐directed and flexible delivery approach, to allow facilitators the opportunity to complete training in their own time, while maintaining an element of peer support through workshops (described below) and the online forum.
Content		
Training content needs to include more focused training on how to deliver the intervention flexibly and in line with the PROGROUP principles, in order to achieve the core function of the intervention.	– The content of the new modules described above includes focused training on group management skills, covering strategies for delivering the intervention flexibly, fostering group ownership and a shared social identity in line with the intervention's principles, as well as techniques for managing group‐based activities and handling difficult group situations (see Figure 1). – The content for the first workshop introduces the intervention's underlying theory (SIM:BC) and provides facilitators the opportunity to share experiences of facilitating groups and co‐create strategies for promoting a shared social identity in weight management groups. – In the second workshop, facilitators practice delivering a selection of PROGROUP sessions (educational and group‐building tasks), in line with the intervention's delivery principles. This is followed by personalised feedback from trainers and peers.	The training content was modified to focus more on group management skills and delivering the intervention flexibly in line with the PROGROUP principles, while retaining elements of peer and trainer support and feedback.

## 4 Discussion

This study used mixed‐methods process evaluation data from the PROGROUP fRCT to inform the optimisation of the PROGROUP training programme in a way that is acceptable to HCPs who receive it. Ultimately, by optimising the training programme, the aim is to enhance HCPs' ability to deliver the intervention with fidelity. Overall, the findings from this study showed that the intervention's manualised activities were mostly delivered as designed and that facilitators were highly skilled in delivering the physical activity and dietary educational components of the intervention (e.g., the ‘eat well’ guide [9]). Nonetheless, the findings also revealed variability in how content was delivered across cohorts, suggesting that some facilitators were more able than others to deliver content flexibly and in line with the PROGROUP principles. Facilitators also found it challenging to attend all training days in full, preferring a more flexible and self‐directed approach to learning. In response to these experiences, the training programme was optimised to include more focused training on group management skills and delivering the intervention in line with the PROGROUP principles (to promote fidelity). The training format was also modified to include self‐directed elements, better aligning with facilitators' learning preferences and supporting its acceptability and feasibility.

Assessing fidelity in complex health interventions such as PROGROUP is crucial for ensuring interventions are delivered as intended. However, most complex interventions require adaptation to fit local contexts and to optimise delivery [15]. In group‐based interventions, an added complexity arises from the interactions between group members and facilitators, making adaptation and flexible delivery (e.g., to meet the group's needs) even more critical for maximising the intervention's potential impact. This highlights the importance of training that not only builds confidence in HCPs to adapt the intervention content to meet the group's needs at any given point in time but also ensures that HCPs are equipped to deliver the intervention with fidelity to its core principles (e.g., creating opportunities for group members to show leadership and ownership). In line with recommendations for improving fidelity in health intervention trials [16], this study assessed and synthesised fidelity data alongside facilitators' training experiences to optimise the training programme. This approach ensures that training equips HCPs with the skills and knowledge to deliver the intervention effectively while maintaining its feasibility and acceptability. The findings demonstrate how to balance intervention fidelity with HCPs' learning preferences, a process that could serve as a model for optimising future training programmes for HCPs.

### 4.1 Wider Implications

The optimised training programme aligns with research suggesting that HCPs prefer a mix of online and self‐directed training methods [17], as well as evidence emphasising the importance of training that is grounded in theory and focused on group facilitation skills for those delivering group programmes [18, 19]. With weight management services increasingly adopting group‐based care models [1], a self‐directed, online training programme focused on managing groups for behaviour change could enhance HCPs' ability to deliver other group‐based interventions. This may include programmes where group‐based behaviour change supports a broader care plan, such as those incorporating obesity management medications [20], or preparing patients for bariatric surgery. With the emerging wave of obesity management medications [20], the optimised training programme may be a timely and valuable resource for preparing HCPs to provide wraparound care for weight management, as group‐based interventions could help streamline the time required for HCPs to deliver behavioural support alongside pharmacological treatments for obesity [20].

Beyond supporting behaviour change, groups have the potential to offer therapeutic benefits, including improved well‐being [21]. In the context of weight management, programmes like PROGROUP may not only help build key resources such as motivation and capability, but also provide a safe, supportive space where patients can connect and support one another to overcome shared psychosocial challenges, such as loneliness. This shared understanding could be particularly beneficial for PLWSO, as they are at higher risk of experiencing psychological distress due to weight stigma, including depression, anxiety and loneliness [22, 23, 24, 25, 26, 27, 28]. The potential for social connection and support derived from belonging to a group could also be valuable for other patient groups (e.g., [29]). However, such potential may only be realised to the extent that HCPs manage and deliver group interventions effectively. Training to provide HCPs with the necessary skills and knowledge to deliver groups with fidelity could thus maximise the impact of group‐based care and potentially enhance the sustainability of group‐based programmes across different health contexts.

Training opportunities for HCPs in health systems such as the NHS are often limited by time and financial constraints. Ongoing pressures, such as staff shortages and the growing need for training opportunities [30], suggest that more flexible training formats, such as online and self‐directed learning, may be better suited to the changing environments of healthcare settings. With services investing more in digital technology to support their workforce [31] and shifting towards more remote and online training (e.g., e‐learning for healthcare [32]), the training programme developed here offers a potentially novel and valuable training opportunity to complement existing training for HCPs delivering group‐based care for PLWSO and potentially for other health conditions as well.

To respond to evolving modalities of care, as some NHS weight management services transition to hybrid or online models of care, training for HCPs should also incorporate strategies that equip practitioners to develop a shared social identity amongst patients in virtual group settings. Using evidence‐based approaches, such as social communication strategies [33], could help promote group cohesion and maintain the therapeutic benefits of group‐based care in these emerging delivery contexts; however, further research is needed to determine how best to optimise online group‐based interventions for PLWSO [33, 34].

### 4.2 Strengths and Limitations

Limitations of this study include the small number of HCPs trained (*N* = 5), which may have limited the breadth of data on training and delivery experiences. Additionally, only a selection of video recordings was analysed, possibly restricting the assessment of delivery fidelity to the PROGROUP principles. Self‐assessed checklists can also introduce self‐report bias; however, no major discrepancies were found between self‐report checklists and in‐person observations. This study also has several notable strengths, including the synthesis of multiple data sources to inform modifications to the training programme. This resulted in a programme that supports the delivery of group‐based interventions, while remaining feasible and acceptable to HCPs.

## 5 Conclusion

This study demonstrates a process for optimising training for HCPs delivering a group‐based behaviour change intervention for PLWSO. The worked example presented here is intended to be generalisable to other healthcare contexts where group‐based care is planned. The findings suggest that, for such interventions to be delivered with fidelity and achieve their intended outcomes, HCPs must develop the necessary skills and knowledge to manage and deliver groups according to the intervention's delivery principles. To ensure feasibility and acceptability, the training format should also be flexible and self‐directed to better accommodate HCPs' schedules and learning preferences. The optimised training programme balances HCPs' preferences with the needs of the intervention and offers a valuable training opportunity for those delivering group‐based care.

## Author Contributions

**Shokraneh Moghadam:** writing – original draft, writing – review and editing, investigation, formal analysis. **Mark Tarrant:** conceptualisation, writing – review and editing, methodology, funding acquisition, supervision, writing – original draft. **Lily Hawkins:** writing – review and editing, investigation, formal analysis. **Dawn Swancutt:** funding acquisition, investigation, writing – review and editing. **Rod Sheaff:** writing – review and editing, funding acquisition. **Laura Hollands:** writing – review and editing, formal analysis, writing – original draft. **Raff Calitri:** conceptualisation, writing – review and editing, investigation, formal analysis, writing – original draft. **Jonathan Pinkney:** conceptualisation, funding acquisition, investigation, writing – review and editing. **Jenny Lloyd:** writing – review and editing, formal analysis, supervision, investigation, funding acquisition.

## Disclosure

The views expressed are those of the author(s) and not necessarily those of the NIHR or the Department of Health and Social Care.

## Ethics Statement

This study has been approved by the NHS Research Ethics Committee (UK REC approval number 21/SW/0144).

## Conflicts of Interest

The authors declare no conflicts of interest.

## Supporting information

Supplementary Information 1 (PROGROUP Training Outline).

Supplementary Information 2 (PROGROUP optimisation schedule).

Supplementary Information 3 [COREQ (COnsolidated criteria for REporting Qualitative research) Checklist].

## Appendix A Fidelity delivery checklist for the PROGROUP principles—example template

**Table:**

SITE ID:	SESSION NUMBER:	DATE:	GROUP ID IF SITE RUNS MORE THAN 1 GROUP:
**FACILITATOR**			
Please comment on the extent to which the facilitator carried out the following behaviours within the entirety of this session (i.e., from beginning to end):			
1. **Promoted** a positive group atmosphere (e.g., welcomed participants, use of humour and friendly language, provided clarification)			
2. **Rewarded** and recognised members' contributions (e.g., congratulated individuals and/or group on achievements, praised individuals and/or group contributions)			
3. **Organised** the group to proactively prevent negative interactions (e.g., identified and agreed to group rules, negotiated and managed group roles and expectations)			
4**. Gave** opportunities for members to show leadership (e.g., encouraged group members to take the lead in group discussions/tasks)			
5. **Related** individual experiences between group members (e.g., emphasised similarities between group members, encouraged group problem solving)			
6. **Used** inclusive language like ‘us’ and ‘we’			
7. **Provided** opportunities for sharing experiences (e.g., invited group members to share experiences, asked for group and/or individual feedback)			
**Additional Notes (e.g., contextual detail)**			

## Appendix B PROGROUP Feasibility RCT Facilitator Interview Topic Guide

*Introductions and confirmation of the consent process and the voluntary nature of the research*.

1 **Background**
  *Aims: To get the facilitator talking and to provide some context for subsequent questions/responses*.
  Could you tell me a little about your history of involvement in weight management programmes
  Probe on facilitation experience, role within the service.
2 **Experiences of PROGROUP training**
  *Aims: To understand how acceptable the training programme is for facilitators—their engagement with the content and their confidence to deliver PROGROUP*.
  Could you tell me how you found the PROGROUP training?
  Probe on how they felt before, during and after the training and their experience of the content/activities/delivery.
3 **Experiences of delivering PROGROUP**
  *Aims: To explore aspects of delivery—what worked, what didn't work so well, delivery challenges, how challenges were overcome and so forth*.
  Could you tell me how the delivery of PROGROUP went for you?
  Probe whether experiences changed over time (beginning, middle and end), what worked, what was challenging, how they overcame these and management of group processes.
  Could you tell me if there were any staffing issues in relation to delivering PROGROUP?
  Probe whether they also delivered usual care, whether the commitment to delivering PROGROUP took them away from other duties, and, if so, the impact of this.
  Could you tell me how you think the participants responded to PROGROUP?
  Probe levels of engagement, attrition
4 **Acceptability of Trial Processes**
  *Aims: To understand the facilitator's experience of being involved in the trial*.
  Could you tell me how you felt about being involved in the trial?
  Probe the quality of interactions with colleagues/researchers, fears/concerns/issues and whether they were sufficiently allayed.
  How did you feel about completing checklists on the content you delivered each session?
  Probe whether they actually did this, when they completed the forms and how much of a burden these were.
  How did you feel about having your session audio/video recorded?
  Probe whether this impacted the delivery of the session in any way.
5 **Contamination**

*Aims: To ascertain if facilitators who also deliver usual care import aspects of PROGROUP*.

Did you use aspects of PROGROUP when you delivered usual care? If so, what were they?

Probe which aspects and why they chose these.

6 **Suggestions for improving PROGROUP and the wider service**

*Aim: To support the optimisation of PROGROUP and the Tier 3 WM service*

What would you change about PROGROUP to maximise the support for change that patients receive?

Probe why these refinements would help

What would you change about training to help you deliver PROGROUP?

Probe why these refinements would help

Do you have any comments/thoughts about how the wider service could be improved?

Would you like to share anything else about your experiences?

## Appendix C Fidelity delivery checklist for PROGROUP manualised content—group session 1 example template

**Table:**

*SITE ID:*	*FACILITATOR NAME:*	*DATE*:			*GROUP ID IF SITE RUNS MORE THAN 1 GROUP*:
**SESSION 1—TEAMWORK MAKES THE DREAM WORK**		**PLEASE TICK**			**IF NOT DELIVERED OR PARTLY DELIVERED, PLEASE GIVE A BRIEF REASON FOR NOT DELIVERING**
		***FULLY DELIVERED***	***PARTLY DELIVERED***	***NOT DELIVERED***	
**START OF SESSION**	1. **Welcome and Housekeeping:** Did you? – Provide individual welcome on arrival?				
**KNOWLEDGE AND ACTIVITIES**	2. **Group Formation—Ice Breakers:** Did you? – Highlight any similarities between members?				
	3. **Outline Group Philosophy**: Did you? – Explain the philosophy/aims of PROGROUP and its structure?				
	4. **Group Agreement**: Did you? – Highlight similarities between members? – Discuss group rules and gain group feedback?				
	5. **What Makes Managing Weight Difficult**: Did you? – Draw on group similarities?				
	6. **Session Break:** Did you? – Mix with members and facilitate conversation? – Focus on group members who might not be engaging?				
	7. **What Contributes to Weight Gain:** Did you? – Explain the key points about PROGROUP and about making changes?				
**END OF SESSION**	8. **End of Session:** Did you? – Outline homework tasks and ask the group to shout‐out take‐home messages?				
**GENERAL COMMENTS:** Please write any general comments you have about today's session in the box below					

## Appendix D Study objectives and outcome measures

**Table:**

Study objectives and linked outcome measures	
**Study objectives**	**Outcome measures**
1. Assess the feasibility and acceptability of the training programme	Semi‐structured interviews
2. Assess intervention delivery fidelity	Fidelity checklists for i. content delivered (completed by facilitators) and ii. PROGROUP principles demonstrated in the delivery of the intervention (completed by researchers). These assessments were based on observational data (video/audio and in‐person) of session delivery.
