# Supplementary material for: Training Healthcare Professionals to Deliver a Group‐Based Intervention for People Living With Severe Obesity: Lessons From the PROGROUP Feasibility Trial
Source: J Hum Nutr Diet. 2026 Jan 28;39(1):e70204. doi: 10.1111/jhn.70204 (PMC12852526; doi:10.1111/jhn.70204)
Supplement: Supplementary file 1 — Supplementary Information 1 (PROGROUP Training Outline). [file JHN-39-0-s001.docx]

**Supplementary Information 1: PROGROUP Training Outline**

**Day 1**

| Welcome & housekeeping |
| --- |
| Introductions |
| Description of PROGROUP  Q&A  Pre-course reading & quiz  Any questions/points to be clarified |
| *Coffee Break* |
| Overview of patient journey  Practical elements of the programme   - Beginning each session - Breaks - Ending of each session - 3Rs |
| Brief intro  More about the training  Group agreement  Key skills and strategies needed for delivery of PROGROUP  Introduce demonstrations  Demos and feedback  Applying to group settings |
| *Lunch* |
| Read & discuss sessions plan/s |
| Feedback & discussion |
| Demonstration: What makes managing weight so important/difficult? |
| De-brief & discussion |
| Demonstration: What contributes to weight gain? |
| De-brief & discussion |
| *Tea* |
| Demonstration: How to manage our weight? [1] |
| De-brief & discussion |
| *Comfort Break* |
| Demonstration: How to manage our weight? [2] |
| De-brief & discussion |
| Q&A, discussion  Reflections on day 1 & looking ahead to day 2  Homework |
| When things go wrong |
| *Finish* |

**Day 2**

| Welcome & housekeeping |
| --- |
| Briefly re-visit group agreement |
| Reflections on day 1 |
| Group pulse |
| A chaotic day |
| De-brief & discussion |
| *Comfort Break* |
| Develop a plan |
| De-brief & discussion |
| How might change affect others? |
| De-brief & discussion |
| *Coffee* |
| Participants log back in |
| Drinks |
| De-brief & discussion |
| *Physical Activity Specialist* |
| Benefits of being more active |
| Reading labels |
| De-brief & discussion |
| Review of the morning session |
| *Lunch* |
| Participants log back in |
| Starchy foods |
| De-brief & discussion |
| Fruit & veg |
| De-brief & discussion |
| *Comfort Break* |
| Strengthening motivation to monitor |
| De-brief & discussion |
| *Tea* |
| Participants log back in |
| *Team 2* |
| Meat, fish & alternatives |
| De-brief & discussion |
| Fats & oils |
| De-brief & discussion |
| *Stretch* |
| How much activity do we need?  What gets in the way of being more active? |
| De-brief & discussion |
| Reflections, looking ahead to day 3 & homework |
| *Finish* |

**Day 3**

| Welcome & housekeeping |
| --- |
| Reflections on Day 2 |
| Group pulse |
| De-brief & discussion |
| Preparation time |
| Eating when not hungry |
| De-brief & discussion |
| Behaviour Chain |
| De-brief & discussion |
| Noticing negative self-talk |
| De-brief & discussion |
| *Coffee Break* |
| Dairy |
| De-brief & discussion |
| Building healthy lunches and main meals |
| De-brief & discussion |
| Eating more mindfully |
| Healthy snacks |
| Debrief & review of morning session |
| *Lunch* |
| Participants log back in |
| Demo: Social network mapping activity |
| De-brief & discussion |
| Strategies for overcoming negative self-talk |
| De-brief & discussion |
| Thinking traps |
| *Tea* |
| De-brief & discussion |
| Meal planning |
| De-brief & discussion |
| *Stretch* |
| Smart shopping |
| De-brief & discussion |
| Reflections |
| Looking ahead to day 4 & homework |
| Take away messages |
| *Finish* |

**Day 4**

| Welcome & housekeeping |
| --- |
| Reflections on day 3 |
| Meeting less frequently [Week 8] |
| De-brief & discussion |
| Preparation Time |
| Managing lapses |
| De-brief & discussion |
| Eating away from home |
| De-brief & discussion |
| *Coffee Break* |
| Participants log back in |
| Managing exercise & eating |
| Activity Thinking traps |
| De-brief & discussion |
| Eating when not hungry |
| Review of morning session |
| *Lunch* |
| Participants log back in |
| Group pulse |
| De-brief & discussion |
| Eating away from home |
| De-brief & discussion |
| *Tea* |
| Participants log back in |
|  |
| Coping with stress |
| De-brief & discussion |
| Sleep & weight management |
| De-brief & discussion |
| Chair based exercises |
| Revisiting my activity plan |
| De-brief & discussion |
| *Stretch* |
|  |
| Week 11 & 12 preparation |
| De-brief & discussion |
| Reflections & looking ahead to day 5 catch up |
| Take away messages |
| *Finish* |

**Day 5 catch up**

| Welcome & housekeeping |
| --- |
| Reflections |
| Social network maps |
| De-brief & discussion |
| Managing slip ups |
| De-brief & discussion |
| Looking to the future |
| De-brief & discussion |
| *Coffee Break* |
| Looking back |
| De-brief & discussion |
| Looking forward |
| De-brief & discussion |
| Coming to the end |
| Review of morning session |
| *Lunch* |
| Dealing with challenges |
| *Tea* |
| FAQs |
| *Stretch* |
| Developing team plans for delivering sessions |
| Reflections & evaluation |
| Take away messages |
| *Finish* |
