# Supplementary material for: Training Healthcare Professionals to Deliver a Group‐Based Intervention for People Living With Severe Obesity: Lessons From the PROGROUP Feasibility Trial
Source: J Hum Nutr Diet. 2026 Jan 28;39(1):e70204. doi: 10.1111/jhn.70204 (PMC12852526; doi:10.1111/jhn.70204)
Supplement: Supplementary file 2 — Supplementary Information 2 (PROGROUP optimisation schedule). [file JHN-39-0-s003.pdf]

PROGROUP optimisation schedule

No.	Category	Key point	Impact on group processes	Data source	Changes suggested by the research team (researcher initials)	Prioritisation (green, amber red) N.B. See definitions	Prioritisation justification (e.g., evidence, feasibility of implementation, impact on logic model)	Where changes needed	Researcher (initials) responsible for making change	
1	Facilitator training/delivery	More training on delivering the intervention flexibly and in line with the PROGROUP principles.	Supports the development of shared social identity between group members.	Facilitator interviews.	Provide more focused training on delivering the intervention in line with the PROGROUP principles.		Supports Mechanism 4: 'Training gives facilitators the skills and knowledge'	Training content.	SM	
2	Facilitator training	Facilitators having all the content prior to the training days to get a feel for the entire proposed programme.	N/A	Facilitator training feedback form.	Provide facilitator handbook and manual ahead of training sessions to read through.		Supports Mechanism 4 (Training gives facilitators the skills and knowledge)	Minor changes to training content.	SM	
3	Facilitator training	More time for each site to prepare for delivery of their sections on the training days.	N/A	Facilitator training feedback form.	As above, provide facilitator handbook and manual ahead of training sessions to read through.		Supports Mechanism 4 (Training gives facilitators the skills and knowledge)	Minor changes to training content.	SM	
4	Facilitator training/delivery	Less hours spent online in training and options for more flexible/self-directed learning to complete in own time.	N/A	Facilitator interviews.	Provide flexible opportunities for facilitators to complete aspects of training in their own time to better suit their availability.		Supports Mechanism 4 (Training gives facilitators the skills and knowledge)	Training format.	SM	
Green	Change deemed necessary and achievable with resources available									
Amber	Change desired, but no resource or time to do it									
Red	Change suggested, but conflicts with other evidence or compromises the logic model									
